# Immunoresponsive Tissue-Engineered Oral Mucosal Equivalents Containing Macrophages

**DOI:** 10.1089/ten.tec.2021.0124

**Published:** 2021-08-18

**Authors:** Bethany Ollington, Helen E. Colley, Craig Murdoch

**Affiliations:** School of Clinical Dentistry, University of Sheffield, Sheffield, United Kingdom.

**Keywords:** oral mucosa, tissue engineering, macrophage, infection, immune response

## Abstract

Macrophages play a key role in orchestrating the host immune response toward invading organisms or non-self molecules in the oral mucosa. Three-dimensional (3D) oral mucosal equivalents (OME) containing oral fibroblasts and keratinocytes are used extensively to mimic the human oral mucosa where they have been employed to examine innate immune responses to both bacterial and fungal pathogens as well as to biomaterials. Although the presence of immune cells is critical in generating an immune response, very few studies have incorporated leukocytes into OME, and to date, none have contained primary human macrophages. In this study, we report the generation of an immunocompetent OME to investigate immune responses toward bacterial challenge. Primary human monocyte-derived macrophages (MDM) were as responsive to bacterial lipopolysaccharide (LPS) challenge when cultured within a 3D hydrogel in terms of proinflammatory cytokine (IL-6, CXCL8, and TNF-α) gene expression and protein secretion compared with culture as two-dimensional monolayers. MDM were incorporated into a type 1 collagen hydrogel along with oral fibroblasts and the apical surface seeded with oral keratinocytes to generate an MDM-containing OME. Full-thickness MDM-OME displayed a stratified squamous epithelium and a fibroblast-populated connective tissue containing CD68-positive MDM that could be readily isolated to a single-cell population for further analysis by collagenase treatment followed by flow cytometry. When stimulated with LPS, MDM-OME responded with increased proinflammatory cytokine secretion, most notably for TNF-α that increased 12-fold when compared with OME alone. Moreover, this proinflammatory response was inhibited by pretreatment with dexamethasone, showing that MDM-OME are also amenable to drug treatment. Dual-labeled immunofluorescence confocal microscopy revealed that MDM were the sole source of TNF-α production within MDM-OME. These data show functional activity of MDM-OME and illustrate their usefulness for investigations aimed at monitoring the immune response of the oral mucosa to pathogens, biomaterials, and for tissue toxicity and anti-inflammatory drug delivery studies.

**Impact statement:**

Three-dimensional *in vitro* models of the oral mucosa have been used extensively to investigate the host response to pathogens, but, to date, few have contained primary leukocytes. In this report, we describe the successful incorporation of primary human macrophages into oral mucosal equivalents (OME). These macrophage-containing models were histologically similar to the oral mucosa and immunoresponsive to bacterial lipopolysaccharides by upregulation of key proinflammatory markers. These advanced OME will significantly aid research into host–pathogen interaction, biomaterial toxicity, and drug delivery studies where the presence of an immune cell component is critical to better represent host oral tissue.

## Introduction

Tissue-engineered oral mucosal equivalents (OME) have been used extensively to study the oral mucosa as improved model systems compared with *in vitro* cultured oral keratinocytes grown as two-dimensional (2D) monolayers.^1^ OME can be in the form of a reconstituted human epithelium where keratinocytes alone are cultured on a porous membrane or as full-thickness cultures that are composed of a fibroblast-populated connective tissue topped with a stratified squamous oral epithelium. Collectively, these OME have been used in numerous studies to study, among others, oral mucosal microbial infection,^2,3^ wound healing,^4,5^ cancer progression,^6,7^ and oral mucositis,^8,9^ as well as to examine the response of the oral mucosa to biomaterials and to monitor toxicity, drug delivery, and efficacy.^10–12^

Resident and recruited immune cells are essential for maintenance of oral tissue and are critical in driving host responses to external insults, while dysregulation of the immune response can cause chronic conditions leading to debilitating oral lesions or poor outcomes in the case of oral squamous cell carcinoma.^13–15^ Macrophages are key innate immune cells found in virtually all tissues. These leukocytes directly protect from invading foreign organisms by phagocytosis, presenting antigens to T lymphocytes, and secreting an array of inflammatory factors that orchestrate the immune response. Macrophage activation can occur through recognition of pathogen-associated molecular patterns, for example, in response to lipopolysaccharides (LPS) of gram-negative bacteria by cell surface pattern recognition receptors, such as CD14 and Toll-like receptors. This interaction induces intracellular signaling leading to increased gene expression and secretion of proinflammatory cytokines such as TNF-α^16^ that can be inhibited by anti-inflammatory therapeutics such as glucocorticoids.^17^ Addition of immune cells, such as macrophages, to current OME would increase their sensitivity to detect and respond to foreign molecules, making them more representative of the native oral mucosa.

Previous studies have generated immune oral gingival models by incorporating primary monocytes, peripheral blood mononuclear cells, or myeloid cancer cell lines (MonoMac-6 [MM6], U937, or THP-1 cells) and by observing changes in inflammatory markers and proteases in response to bacterial LPS,^18–21^ bacterial biofilms,^22^ or X-ray treatment.^23^ While the use of myeloid cancer cell lines presents fewer technical limitations and reproducibility compared with primary immune cells, there is good evidence that their phenotype and function is markedly altered compared with primary cells, with the often used THP-1 cells shown to express aberrant levels of key macrophage phenotypic markers and thus respond differently to stimuli.^24^ Moreover, peripheral blood monocytes very rapidly differentiate into macrophages upon crossing the vasculature as they migrate into tissues and so incorporation of macrophages rather than monocytes into OME is more desirable. Therefore, use of primary macrophages is preferential for use in *in vitro* OME as these cells better represent the innate immune component of human tissue.

Here, we describe the generation of a tissue-engineered immune model of nonkeratinized oral mucosa containing primary human monocyte-derived macrophages (MDM). These immune OME produced functional responses upon stimulation with *Escherichia coli* LPS in the form of increased secretion of proinflammatory cytokines that were inhibited by dexamethasone treatment. These OME will be extremely beneficial for studies aimed at investigating macrophage behavior in disease and cellular toxicity, as well as for examining immune response to drug and/or biomaterials exposure.

## Methods

### Materials

Unless otherwise stated, all reagents were purchased from Sigma–Aldrich, United Kingdom.

### Cell isolation and culture

MDM were differentiated from primary human monocytes as previously described.^25^ Briefly, buffy coats obtained from the National Blood Service, United Kingdom (Ethical Approval No. 012597) were diluted 1:1 in Hank's balanced salt solution (without Ca^2+^ and Mg^2+^) and mononuclear cells separated by density-gradient centrifugation using Ficoll^®^ Paque Plus (GE Healthcare, United Kingdom). Monocytes were purified by plastic adherence and differentiated to MDM by 7 days culture in Iscove's modified Dulbecco's medium supplemented with 2% human AB serum, 2 mM l-glutamine, 100 IU/mL penicillin, and 100 μg/mL streptomycin. Differentiation of monocyte to MDM was confirmed by cell morphology, gene expression, and cell surface protein abundance of key differentiation markers.

FNB6-hTERT immortalized oral keratinocytes (Ximbio, United Kingdom)^26^ were cultured in flavin- and adenine-enriched medium consisting of Dulbecco's modified Eagle's medium (DMEM) and Ham's F12 medium in a 3:1 (v/v) ratio supplemented with 10% (v/v) fetal bovine serum (FBS), 10 ng/mL epidermal growth factor, 0.18 mM adenine, 5 μg/mL insulin, 5 μg/mL transferrin, 2 mM L-glutamine, 0.2 nM triiodothyronine, 0.625 μg/mL amphotericin B, 100 IU/mL penicillin, and 100 μg/mL streptomycin. Normal oral fibroblasts (NOF) were isolated from the connective tissue of biopsies obtained from the oral mucosa of patients during routine dental procedures with written informed consent (Ethical Approval No. 09/H1308/66) and cultured in DMEM supplemented with 10% v/v FBS, 2 mM L-glutamine, 100 IU/mL penicillin, and 100 μg/mL streptomycin.^6^

### Generation of MDM-OME

OME were constructed as previously described.^27^ In brief, NOF were mixed with rat tail type I collagen (2 × 10^5^ NOF/mL collagen), and 1 mL aliquoted into 12 mm cell culture transwell inserts (0.4 μm Polyethylene Terephthalate; Merck Millipore, USA) and the cell-populated collagen hydrogels incubated in a humidified atmosphere at 37°C for 2 h to solidify. FNB6-hTERT cells (5 × 10^5^) were then seeded on the surface of the collagen hydrogel and cultured submerged for 2 days, after which the models were raised to an air-to-liquid interface and cultured for a further 10 days. To generate MDM-OME, 7-day MDM (1 × 10^6^ MDM/OME) were added to collagen hydrogels alongside NOF. A single batch of collagen was used in all experiments that contained <0.2 EU/mL endotoxin when quantified by limulus amebocyte lysate assay (Thermo Fisher Scientific, United Kingdom).

### Simulation of MDM and MDM-OME

LPS from *E. coli* 0111:B4 (500 ng per 1 × 10^6^ MDM; Invivogen, USA) was diluted in culture medium and used to treat MDM for 24 h to stimulate an inflammatory response. Where indicated, MDM were pretreated for 4 h with 1 μg/mL dexamethasone (Abcam, USA) diluted in culture medium before LPS stimulation to inhibit the inflammatory response. MDM or MDM-OME incubated with medium alone were used as unstimulated controls.

### Protein secretion and gene expression analysis

Following 24 h incubation with LPS, secretion of the inflammatory cytokines TNF-α, CXCL8, and IL-6 into conditioned media were quantified by ELISA (Human Duoset; R&D systems, USA) and cell toxicity measured by release of lactate dehydrogenase (CytoTox96; Promega, United Kingdom). Total RNA was extracted following the manufacturer's instructions (Monarch Total RNA Miniprep Kit; New England Biolabs, United Kingdom) and then reverse transcribed to cDNA (High-Capacity RT Kit; Thermo Fisher Scientific), with the amount of RNA used kept consistent within experiments. Quantitative polymerase chain reaction (qPCR) was performed with TaqMan gene expression assays using VIC-labeled β2-microglobulin probe (hs00187842_m1) as a reference control alongside FAM-labeled target probes for: *CD11c* (hs00174217_m1), *CD14* (hs02621496_s1), *CD36* (hs01567185_m1), *CD80* (hs00175478_m1), *CD163* (hs00174705_m1), *CD206* (hs00267207_m1), *carboxypeptidase M (CPM)* (hs01074151_m1), *HLA-DRA* (hs00219575_m1), *vimentin* (hs00958111_m1), *CXCL8* (hs00174103_m1), *IL-6* (hs00174131_m1), and *TNF-α* (hs01113624_g1). Expression of the target gene was normalized to the abundance of the control gene transcript, *β-2-microglobulin.*

### Flow cytometry

MDM cultured as monolayer were removed from the culture dish using a cell scraper and resuspended at 5 × 10^5^ cells/mL in FACS buffer (phosphate-buffered saline [PBS], pH 7.2; 0.1% bovine serum albumin; 0.1% sodium azide). Cells were incubated on ice for 5 min in FACS buffer containing 5 μL Fc blocking reagent (Miltenyi Biotech, Germany) and then incubated for 20 min at 4°C in the dark with one of the following allophycocyanin (APC)-conjugated antibodies: Mouse IgG control (17-4714-81; eBioscience, USA), CD11c (130-114-110; Miltenyi Biotech), CD14 (17-0149-42; eBioscience), CD36 (130-095-475; Miltenyi Biotech), and HLA-DR (130-111-943; Miltenyi Biotech). MDM-OME were digested by 1 h incubation with 2 mg/mL collagenase type 1 (Life Technologies, UK) at 37°C and passed through a cell strainer (Corning, UK) to obtain single-cell suspensions. The LIVE/DEAD flexible blue stain (Thermo Fisher Scientific) was used, as per manufacturer's instructions, to measure and exclude dead cells from the analysis and APC-conjugated CD11c antibody used to identify MDM. Flow cytometry was performed using an LSRII (BD Biosciences, USA) and analyzed by the FlowJo software v10.7 (BD Biosciences).

### Histological and immunohistochemical analysis

MDM-OME were washed with PBS, fixed in 10% neutral buffered formalin for 24 h, and paraffin-wax embedded using standard histology procedures. Sections (5 μm) were cut using a Leica RM2235 microtome (Leica Microsystems, Germany) and mounted on Superfrost Plus slides (Thermo Fisher Scientific). Sections stained with hematoxylin and eosin were mounted in dibutylphthalate polystyrene xylene and imaged using an Olympus BX51 microscope with cellSens Imaging Software (Olympus GmbH, Germany). For immunofluorescence staining, sections were dewaxed, dehydrated, and high**-**temperature Tris/EDTA buffer (pH 9) antigen retrieval performed, then blocked with goat serum for 20 min. Sections were incubated with mouse anti-human CD68 (0.4 μg/mL; clone KP1, Abcam ab955) followed by fluorescein isothiocyanate-conjugated goat anti-mouse F(ab) IgG (1 μg/mL, Abcam ab6669) and recombinant rabbit anti-human TNF-α (8 μg/mL, 17590-1-AP, Proteintech, UK) followed by Cy3-conjugated donkey anti-rabbit IgG (Jackson Immunoresearch, USA) for 1 h at room temperature, with PBS washing between incubations. After staining, slides were mounted with Prolong Diamond anti-fade (Thermo Fisher Scientific), cured for 24 h at room temperature in the dark and imaged using a Zeiss Axioplan 2 microscope with the Image ProPlus v7.0.1 software (Media Cybernetics Inc., MD, USA).

### Statistical analysis

All data are presented as mean ± standard deviation of at least three independent biological experiments. Statistical analysis was undertaken using GraphPad Prism (v9.0.2, GraphPad Software). Pairwise comparisons were performed using Students *t*-test and group-wise comparisons using one-way analysis of variance followed by Tukey's *post-hoc* multiple comparisons test. Differences between groups was considered significant when *p* < 0.05.

## Experiment

### MDM characterization, and their activation and inhibition in 2D monolayer and three-dimensional collagen hydrogels

Monocytes isolated from peripheral blood were differentiated to MDM in 2D culture as determined by changes to cell morphology, such as increased cell size and nuclear to cytoplasm ratio (Supplementary Fig. S1A, B), and alterations in the gene expression and cell surface protein abundance of key differentiation markers. These included the presence of vimentin and *CD11c* gene expression, increased gene expression of *CPM* and *CD36*, along with decreased gene expression of *CD14*, as the monocytes differentiate to MDM (Supplementary Fig. S1C). In addition, increased cell surface abundance of CD11c, CD36, and HLA-DR as well as decreased abundance of CD14 by MDM compared with monocytes was observed by flow cytometry (Supplementary Fig. S1D), in line with previous reports.^28–30^

LPS isolated from *E. coli* was used to show a functional response of 2D-cultured MDM to bacterial-mediated challenge, whereas the anti-inflammatory glucocorticoid, dexamethasone, inhibited LPS-induced protein secretion of TNF-α, CXCL8, and IL-6 in a dose-dependent manner (Supplementary Fig. S2A–C). Inhibition of cytokine secretion by pretreatment with dexamethasone was significant at concentrations ranging from 0.1 to 100 μg/mL at both 4 and 24 h without affecting MDM cell viability compared with untreated controls (Supplementary Fig. S2A–D). Treatment of MDM with 1 μg/mL dexamethasone for 4 h was used in subsequent experiments.

**FIG. 1.**
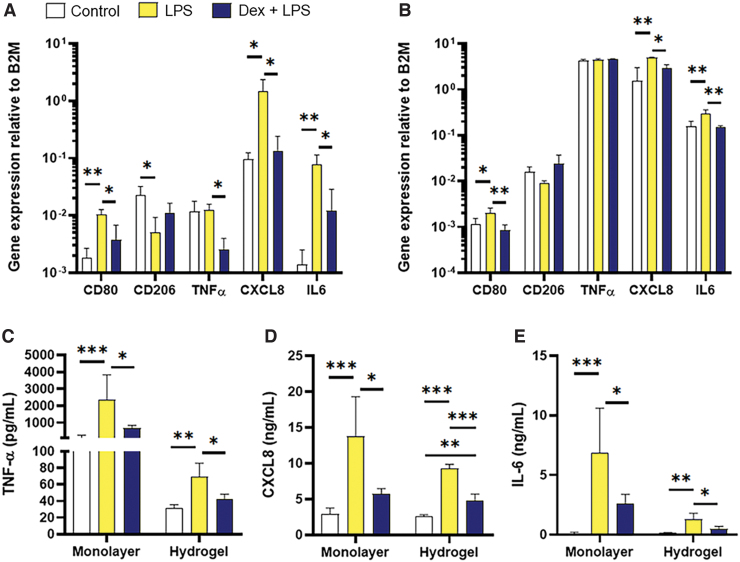
Activation of MDM in monolayer and 3D collagen hydrogels. MDM were cultured as monolayers **(A)** or within 3D collagen hydrogels **(B)** for 24 h before incubation with *E. coli* LPS (500 ng/10^6^ MDM) for 24 h or preincubated with Dex (1 μg/mL) for 4 h before addition of *E. coli* LPS for 24 h, controls received vehicle only for 24 h. Gene expression was analyzed by qPCR for *CD80*, *CD206*, *TNF-α*, *CXCL8*, and *IL-6*, and expression calculated relative to the reference control B2M. Secretion of TNF-α **(C)**, CXCL8 **(D)**, and IL-6 **(E)** was measured by ELISA. Data are presented as mean ± SD with statistically significance differences determined using one-way ANOVA and compared with untreated control. *p < 0.05, **p < 0.01, ***p < 0.005; *n* = 3. 3D, three-dimensional; ANOVA, analysis of variance; B2M, β2-microglobulin, Dex, dexamethasone; *E. coli*, *Escherichia coli*; ELISA, enzyme-linked immunosorbent assay; LPS, lipopolysaccharides; MDM, monocyte-derived macrophages; qPCR, quantitative polymerase chain reaction; SD, standard deviation. Color images are available online.

To determine the MDM function in three-dimensional (3D) as well as 2D, MDM were cultured as monolayer or within a type I collagen hydrogel to mimic the connective tissue component of the oral mucosa. In 2D monolayer cultures, LPS treatment significantly increased gene expression of inflammatory mediators *CD80* (*p* < 0.01), *CXCL8* (*p* < 0.05), and *IL-6* (*p* < 0.01) compared with unstimulated controls (Fig. 1A). Expression of *CD206* was significantly decreased upon LPS stimulation (*p* < 0.05) while that of *TNF-α* remained unchanged. For *CD80, TNF-α*, *CXCL8*, and *IL-6* changes in gene expression were significantly inhibited by pretreatment with dexamethasone (*p* < 0.05; Fig. 1A). In most instances, the functional activity of MDM in 3D culture replicated those observed in 2D. Once again, significantly increased gene expression was observed for *CD80* (*p* < 0.05), *CXCL8* (*p* < 0.01), and *IL-6* (*p* < 0.01) upon LPS stimulation that was inhibited by dexamethasone pretreatment (*p* ≤ 0.05; Fig. 1B). In contrast to 2D cultures, no differences in gene expression were found with *CD206* or *TNF-α* between treated MDM and controls when cultured in 3D hydrogels (Fig. 1B). Analysis of MDM protein secretion upon LPS and dexamethasone treatment was consistently similar for 2D and 3D cultures. Here, secretion of TNF-α, CXCL8, and IL-6 was significantly increased by LPS for 2D (*p* < 0.001) and 3D (*p* ≤ 0.01) cultures, and these increased levels were significantly inhibited back to control levels by pretreatment with dexamethasone (≤ 0.05 for 2D and 3D, Fig. 1C, D). These data suggest that MDM response to LPS or glucocorticoids is retained in a 3D environment similar to that observed in 2D *in vitro* culture.

### Generation and histological validation of MDM-OME

**FIG. 2.**
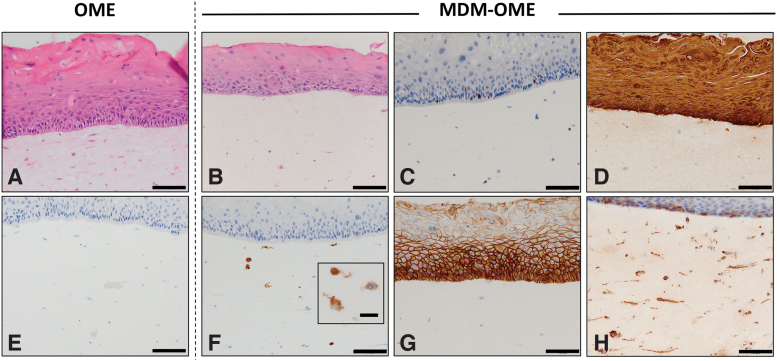
Histological characterization of MDM-OME. Models were cultured for 10 days at air-to-liquid interface and analyzed by histology with hematoxylin and eosin staining **(A, B)** and immunohistochemistry for immunopositive expression of ki67 **(C)** cytokeratin (AE1/3) **(D)**, CD68 **(E, F)**, E-cadherin **(G)**, and vimentin **(H)**. Images presented as representative from at least three independent experiments. Scale bar = 100 μm and 20 μm for the inlay box in (**F**). OME, oral mucosa equivalent. Color images are available online.

Histological analysis of OME and MDM-OME revealed that both models were composed of a nonkeratinized, stratified squamous epithelium consisting of oral keratinocytes that progressively differentiate toward the apical surface, along with a cell-populated connective tissue (Fig. 2A, B). Immunopositive intracellular staining for the well-characterized macrophage marker CD68 identified MDM that were dispersed throughout the connective tissue component of MDM-OME, but not OME (Fig. 2E, F). We have extensively characterized OME previously in terms of their histological markers and ultrastructure and so analysis was not performed in this study.^27,31^ MDM-OME displayed proliferating (ki67^+^) keratinocytes in the basal layer of the epithelium (Fig. 2C) that was also highly immunoreactive to the pan-cytokeratin antibody AE1/3 confirming the presence of differentiating keratinocytes throughout the epithelium (Fig. 2D). Immunopositive E-cadherin staining was observed at the keratinocyte cell–cell junctions, indicating a well-formed epithelial network (Fig. 2G), whereas vimentin was observed in both the fibroblasts and MDM within the connective tissue, as well as within some of the basal keratinocytes (Fig. 2H).

### MDM single-cell analysis and viability within MDM-OME

**FIG. 3.**
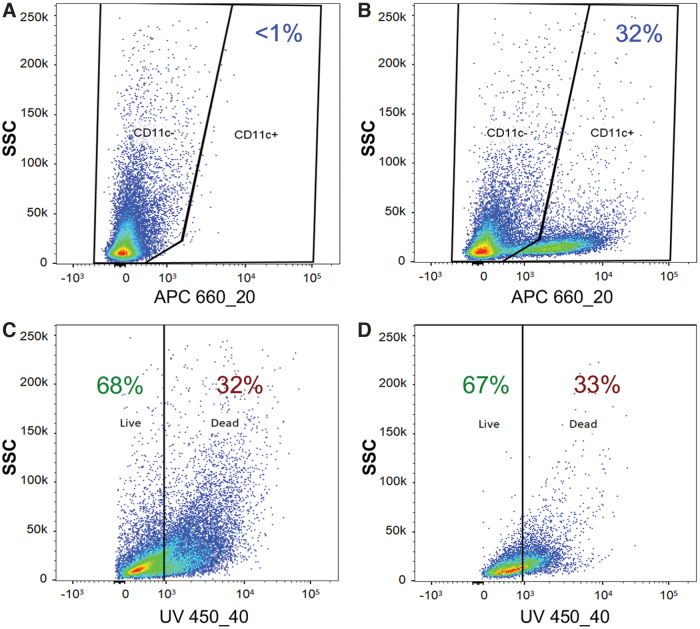
Viability of MDM within MDM-OME. Collagenase-treated OME **(A)** and MDM-OME **(B)** were stained with APC-conjugated anti-CD11c to identify the presence of MDM. Cells identified as CD11c^–^ **(C)** or CD11c^+^ **(D)** were gated and assessed for viability. Graphs are representative of at least three independent experiments. APC, allophycocyanin. Color images are available online.

Following collagenase disaggregation of the hydrogel, an MDM-specific flow cytometric gating strategy was employed to measure the MDM viability within MDM-OME. Cells were dual stained with a fluorescent viability dye and an APC-conjugated monoclonal antibody for pan-monocyte-derived cell marker, CD11c, to specifically identify MDM within the heterogeneous cell population. When segregated into CD11c^–^ and CD11c^+^ cells, MDM-OME contained 32% ± 10% CD11c^+^ MDM, whereas OME contained <1% CD11c^+^ due to nonspecific antibody staining (Fig. 3A, B). The viability of the CD11c^–^ keratinocyte and fibroblast population was 68% ± 4% and was similar to the viability of this cell population in OME (73% ± 4%), whereas the MDM CD11c^+^ viability in MDM-OME was 67% ± 8% (Fig. 3C, D).

### MDM-OME respond to LPS by secretion of TNF-α

**FIG. 4.**
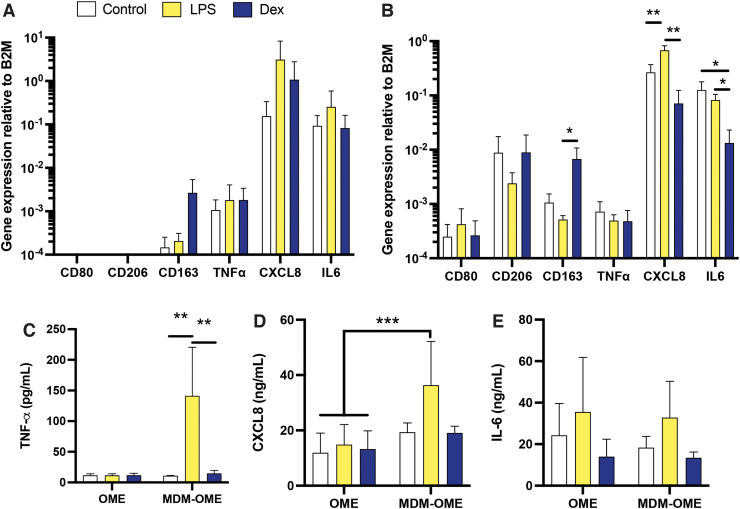
Immune response of MDM within an OME upon challenge with *E. coli* LPS. OME or MDM-OME were challenged with LPS (500 ng/10^6^ MDM) for 24 h following treatment for 4 h with or without Dex (1 μg/mL). Gene expression was analyzed by qPCR for markers of macrophage polarization (*CD80*, *CD206*, and *CD163*) and inflammation (*TNF-α*, *CXCL8*, and *IL-6*), relative to B2M for OME **(A)** and for MDM-OME **(B)**. Proinflammatory cytokine release was analyzed by ELISA for TNF-α **(C)**, CXCL8 **(D)**, and IL-6 **(E)**. Data are presented as mean ± SD with statistically significance differences determined using one-way ANOVA. *p < 0.05, **p < 0.01, ***p < 0.005; *n* = 3. Color images are available online.

To assess the MDM function within MDM-OME, changes in gene expression and inflammatory cytokine secretion were quantified in response to 24 h LPS treatment, with or without 4 h dexamethasone pretreatment. As expected, gene expression of the macrophage-specific markers *CD80* and *CD206* was not detected in OME (Fig. 4A). Gene expression was detected for *CD163*, *TNF-α*, *CXCL8*, and *IL-6*, but levels of expression were similar between treatments for all genes (Fig. 4A). In MDM-OME, gene expression of the macrophage markers *CD80* and *CD206* was observed, but there were no differences between control and treated samples, as was the case for *TNF-α* (Fig. 4B). Pretreatment with dexamethasone significantly increased gene expression of CD163 but only in the presence of LPS (*p* < 0.05). Gene expression for *CXCL8* was significantly increased (*p* < 0.01) in LPS-treated MDM-OME compared with unstimulated controls, and levels of this chemokine were inhibited by pretreatment with dexamethasone (*p* < 0.01; Fig. 4B), whereas mRNA levels of *IL-6* were similar in control and LPS-treated samples but significantly reduced by dexamethasone (*p* < 0.05; Fig. 4B).

**FIG. 5.**
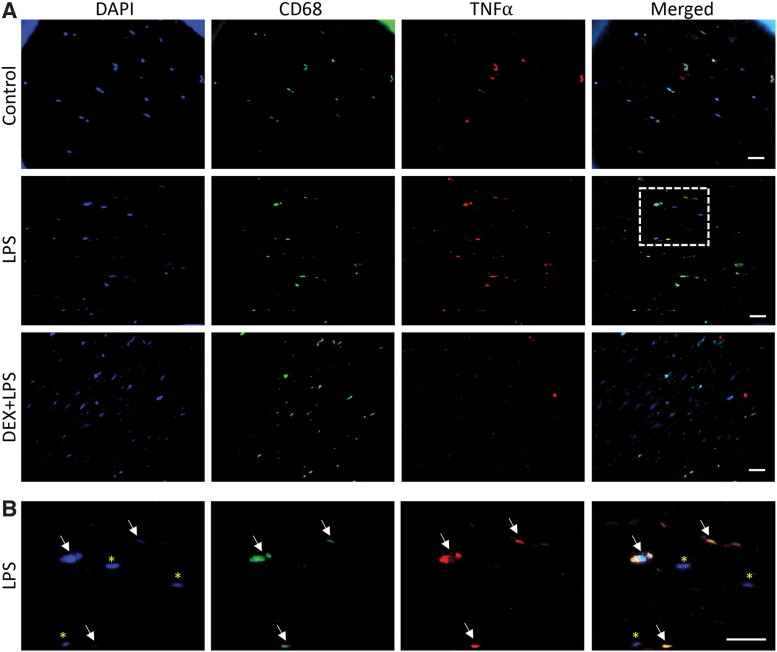
TNF-α secretion by MDM in MDM-OME. MDM-OME were challenged with LPS for 24 h following pretreatment for 4 h with or without 1 μg/mL Dex+LPS; controls received no treatment. Immunofluorescence staining of tissue sections was performed for CD68 (*green*), TNF-α (*red*), counterstained for nuclei with DAPI (*blue*), and images merged **(A)**. Panel **(B)** shows a magnified image of the *white square* displayed in LPS-treated MDM-OME in (**A**). Scale bars = 20 μm. Color images are available online.

The conditioned medium was also assessed for secreted TNF-α, CXCL8, and IL-6 from MDM-OME and compared with OME. No differences in TNF-α were observed in OME between any treatments, whereas a 10-fold increase was observed in TNF-α levels of LPS-treated MDM-OME compared with control MDM-OME that were significantly inhibited by dexamethasone pretreatment (*p* < 0.01, Fig. 4C). CXCL8 released by LPS-treated MDM-OME was significantly (*p* < 0.05) elevated in comparison to all OME irrespective of treatment but were not significantly different from control or dexamethasone-treated MDM-OME (Fig. 4D), whereas IL-6 levels were similar for all samples tested (Fig. 4E). These data suggest that LPS induced a robust TNF-α response but only when MDM were present in the OME. Dual-labeled immunofluorescence microscopy for CD68 and TNF-α confirmed that immunopositive staining for TNF-α was increased in LPS-treated MDM-OME and reduced by dexamethasone (Fig. 5A). Moreover, TNF-α co-localized with that of CD68-positive cells and not NOF in LPS-treated MDM-OME (Fig. 5B), indicating that LPS-mediated TNF-α release within the MDM-OME 3D models is MDM-specific.

## Discussion

The importance of macrophages in initiating an immune response in the oral mucosa as well as their significance in multiple oral diseases is well recognized.^13,14,32^ However, macrophage responses to external stimuli are often examined in isolation to other cells, more often than not as 2D monolayer cultures where cell surface adhesion-matrix contacts are limited. *In vivo* macrophages experience life in a 3D context, therefore developing 3D tissue-engineered *in vitro* models with increasing complexity is of particular importance if we are to fully understand how cells function in native tissue. Although the use of tissue-engineered OME is now relatively common in oral mucosal studies, the incorporation of additional cells into these systems, especially immune cells, is lacking. Here, we describe the construction of tissue-engineered OME that contain functional primary human macrophages.

To date, very few studies have attempted to include macrophages cells into 3D oral mucosal models. The 3D co-culture model developed by Morin and Grenier composed of primary gingival fibroblasts embedded within a collagen hydrogel and overlaid with phorbol 12-myristate 13-acetate (PMA)-stimulated U937 cells that were responsive to *Aggregatibacter actinomycetemcomitans* LPS treatment.^18^ Xiao *et al.* punched a hole in a full-thickness OME composed of primary gingival fibroblasts, topped with the HaCaT skin keratinocyte cell line and injected it with LPS-stimulated, PMA-differentiated THP-1 cells to mimic periodontal disease.^20^ Bao *et al*.^22^ developed a similar periodontal model using HPV E6/7-immortalized gingival fibroblasts, keratinocytes, and MM6 cells to produce an immune model that was then challenged with multispecies bacterial biofilm to mimic subgingival plaque. To date, Holmström *et al.* have developed the most characterized immune-responsive OME. Their OME of gingival inflammation were stimulated with LPS for several days to induce peripheral blood monocyte differentiation *in situ*. Although the OFK6/TERT-2 epithelium displayed a lack of differentiation, as observed in previous publications,^33^ the presence of immune cells within the connective tissue was confirmed by CD68 staining and their OME responded to repeated LPS challenge with increased proinflammatory cytokine expression.^19^

The importance of using primary human-derived macrophages in such *in vitro* models cannot be overstated. Many studies examining macrophages have used monocytic cancer cell lines differentiated by culture with the potent protein kinase C activator, PMA, to mimic these innate immune cells. Although adequate and convenient for some assays, these cell lines often fall short in terms of native phenotypic and functional responses when compared with primary cells. As a typical example, peripheral blood monocytes abundantly express the archetypal cell surface marker and LPS pattern recognition receptor CD14, whose expression is notably decreased when these cells differentiate to MDM. In contrast, undifferentiated THP-1 monocytes express negligible cell surface CD14 but following differentiation to a macrophage morphology by PMA treatment, expression is significantly increased,^34,35^ which affects their cellular response to stimuli.^24^

Primary MDM cultured in a 3D environment responded to *E. coli* LPS in a similar manner to 2D cultured MDM in terms of cell surface marker expression and secretion of proinflammatory cytokines. Moreover, 3D cultured MDM displayed increased expression of the classical LPS-induced M1 macrophage phenotypic markers CD80, CXCL8, and IL-6, with simultaneously decreased expression of the M2 marker, CD206 similar to 2D polarized MDM,^30^ indicating that these cells can be polarized *in situ* within a tissue-like environment. Inhibition of this LPS-induced cytokine response by the well-characterized anti-inflammatory glucocorticoid dexamethasone^36^ additionally indicates that MDM within an *in vitro* stroma are amenable to drug treatment. Interestingly, pretreatment with dexamethasone inhibited *TNF-α* gene transcription in MDM 2D but not in any of the 3D culture systems, whereas TNF-α protein secretion was inhibited to control levels in both 2D and 3D culture systems. Gewert *et al.* previously showed that pretreatment with dexamethasone completely inhibited TNF-α protein secretion in LPS-treated murine macrophages while *TNF-α* mRNA was only partially inhibited.^37^ It is also possible that MDM are less sensitive to dexamethasone and possibly other drug treatments in 3D tissue culture environments than 2D, a phenomenon seen for other 3D culture systems.^38^

Incorporation of MDM into OME did not affect the tissue architecture or histological characteristics of the tissue. CD11c was found to be a reliable marker to separate MDM from keratinocytes and fibroblasts by flow cytometry and in conjunction with viability selection could be used effectively to sort MDM into single-cell populations for further downstream analysis such as establishment of MDM polarization status by multichromatic flow cytometry or single-cell gene expression profiling.

The oral cavity is heavily colonized with more than 500 different species of microorganisms that constitute the commensal oral microbiome,^39^ yet the oral epithelium remains in a quiescent state in health because the epithelium is predominantly nonresponsive to bacterial challenge at the mucosal surface. Indeed, OME exposed to LPS displayed similar levels of cytokine gene expression and protein section to unstimulated controls, as observed previously.^27^ It is the interaction between microbial mediators and innate immune cells that reside in the epithelium or connective tissue that are crucial for driving immunity, which is why incorporation of macrophages into OME is imperative if host pathogen responses are to be replicated *in vitro*.

LPS induced a significant increase in TNF-α protein release in MDM-OME, compared with OME, which was prevented by dexamethasone treatment, although this was not matched by gene expression. This is likely due to the *in vitro* models being sampled at 24 h, a time point where protein secretion is maximal, but *TNF-α* mRNA expression may have returned to baseline levels. Fluorescence imaging showed that TNF-α was almost exclusively secreted from MDM within the collagen connective tissue, underscoring the importance of these innate immune cells in driving the inflammatory response. Once secreted, TNF-α is a key mediator in initiating the immune response by inducing the gene expression and protein secretion of an array of proinflammatory factors by neighboring cells, such as fibroblasts and keratinocytes, which then further orchestrate inflammation. This cascade effect was observed by the increased gene expression of *CXCL8* and *IL-6* in MDM-OME but not OME after 24 h, as the MDM-secreted TNF-α stimulated proinflammatory gene transcription in surrounding cells. Protein secretion of CXCL8, but not IL-6, mirrored this pattern in LPS-stimulated MDM-OME compared with OME alone at 24 h. Cytokine release may increase further over time as gene transcription progresses to translation and protein secretion, as was observed by Holmström *et al*. in their OME of gingival inflammation that was stimulated repeatedly with LPS for several days.^19^

In acute inflammation, levels of TNF-α and other proinflammatory cytokines subside as infection is resolved. However, persistently high levels of TNF-α are observed in several oral diseases such as oral lichen planus,^40^ periodontitis,^19^ and oral squamous cell carcinoma,^41^ where the presence of macrophages is heavily implicated in the disease process. Inclusion of MDM into OME represents an essential component of both a healthy or disease model and will aid in the understanding of how these cells drive disease progression and how the unresolved proinflammatory cascade can be modulated.

In conclusion, MDM are suitable for inclusion in tissue-engineered OME and improve the functional response of these models to inflammatory stimuli, notably the secretion of MDM-derived TNF-α. This improved model system has potential applications in several areas of oral bioscience including oral mucosal responses to microorganisms and analysis of host–pathogen interactions, chronic inflammatory conditions, drug delivery, or adverse reaction to biomaterials.

## Authors' Contributions

C.M., B.O., and H.E.C. conceived the research and designed experiments. B.O. performed the experiments and collected the data. B.O., H.E.C., and C.M. analyzed the data, conducted statistical analysis, and interpreted the results. B.O. wrote the article draft and prepared the figures; further article editing was performed by H.E.C. and C.M. All authors read and approved the article.

## Supplementary Material

Supplemental data

Supplemental data
