# Supplementary material for: Immunoresponsive Tissue-Engineered Oral Mucosal Equivalents Containing Macrophages
Source: Tissue Eng Part C Methods. 2021 Aug 18;27(8):462–71. doi: 10.1089/ten.tec.2021.0124 (PMC8403184; doi:10.1089/ten.tec.2021.0124)

**Supplementary Figure 1: Differentiation of monocytes to monocyte-derived macrophages (MDM).** Monocytes isolated from peripheral blood were seeded in tissue culture plates. Cell morphology was assessed by light microscopy after 24 h showing monocytes **(A)**, and larger differentiated MDM at day 7 **(B)**. Gene expression was analysed by qPCR for *CD11c, CD14, CD36, HLA-DRA,* *carboxypeptidase M* (*CPM*) and *vimentin* (*vim*) for monocytes and MDM, relative to *β2-microglobulin* (B2M) **(C)** and cell surface protein expression measured by flow cytometry for CD11c, CD14, CD36 and HLA-DR. Each marker (black, empty) was normalised to IgG control (blue, filled) to give median fluorescent values **(D)**. Data are presented as mean ± SD with statistically significance determined using one-way ANOVA; *p<0.05, **p<0.01, ***p<0.005; n=3. Scale bars = 100 µm.


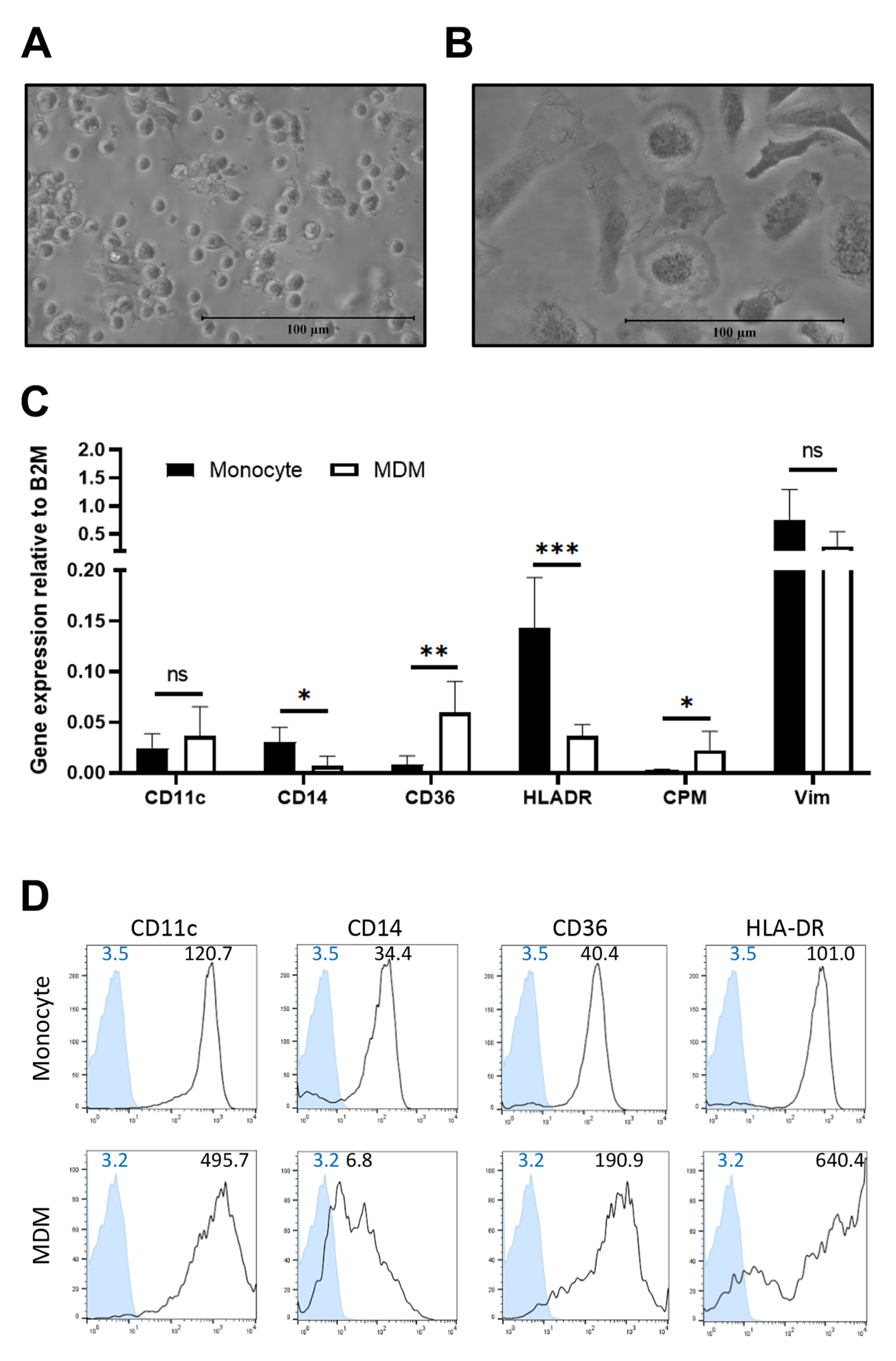

Supplement: Supplemental data [file Suppl_FigureS1.docx]
