# Supplementary material for: Immunoresponsive Tissue-Engineered Oral Mucosal Equivalents Containing Macrophages
Source: Tissue Eng Part C Methods. 2021 Aug 18;27(8):462–71. doi: 10.1089/ten.tec.2021.0124 (PMC8403184; doi:10.1089/ten.tec.2021.0124)

**Supplementary Figure 2: Optimisation of dexamethasone-mediated inhibition of LPS-induced monocyte-derived macrophage (MDM) activation.** MDM were treated with dexamethasone (0.01-100 µg/mL) for 4 h (green) or 24 h (grey, dashed) before treated with *E. coli* LPS (500 ng/10^6^ MDM) for a further 24 h. Pro-inflammatory cytokine release was analysed by ELISA for TNF-α **(A),** CXCL8 **(B)**, and IL-6 **(C)** and lactate dehydrogenase (LDH) release quantified as a marker of cell viability **(D).** Data are presented as mean ± SD with statistically significance differences determined using one-way ANOVA; *p<0.05, **p<0.01, ***p<0.005; n=3.


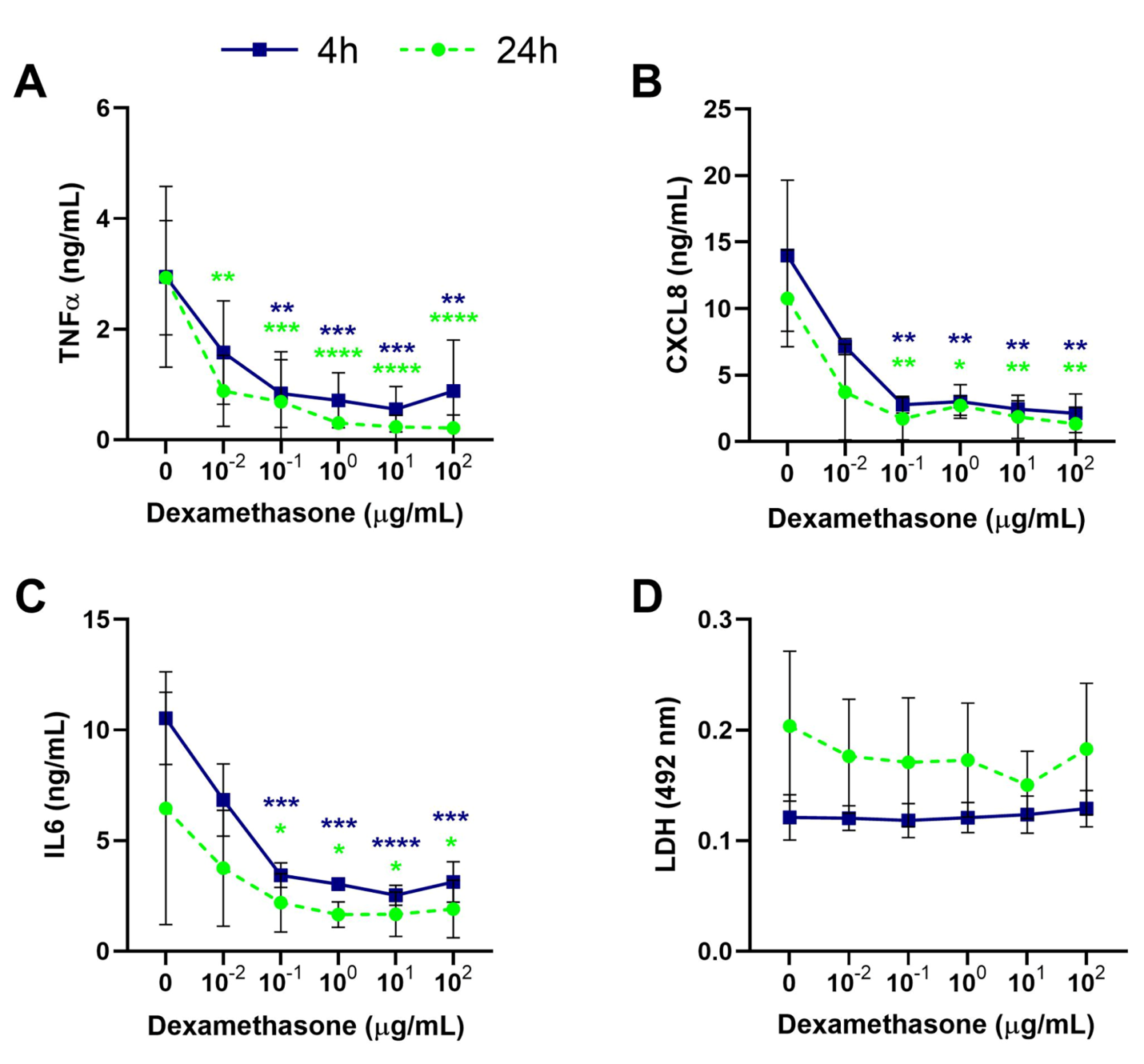

Supplement: Supplemental data [file Suppl_FigureS2.docx]
